# Association of Asian Dust with daily medical consultations for pollinosis in Fukuoka City, Japan

**DOI:** 10.1186/s12199-017-0623-x

**Published:** 2017-04-04

**Authors:** Soyoko Sakata, Shoko Konishi, Chris Fook Sheng Ng, Reiko Kishikawa, Chiho Watanabe

**Affiliations:** 1 Department of Human Ecology, Graduate School of Medicine, The University of Tokyo, 7-3-1 Hongo, Bunkyo-ku, Tokyo, 113-0033 Japan; 2 Department of Anthropology, University of Washington, Box 353100, Seattle, WA 98195-3100 USA; 3 Department of Pediatric Infectious Diseases, Institute of Tropical Medicine, Nagasaki University, 1-12-4 Sakamoto, Nagasaki City, Nagasaki 852-8523 Japan; 4 Clinical Research Center, Fukuoka National Hospital, 4-39-1 Yakatabaru, Minami-ku, Fukuoka 811-1394 Japan

**Keywords:** Asian Dust, Particulate matter, Pollen, Pollinosis, Air pollution

## Abstract

**Background:**

The objective is to examine the association between AD and the daily number of medical consultations for pollinosis in Fukuoka City.

**Methods:**

We analyzed 65,488 daily medical consultations for pollinosis from 4 clinics in Fukuoka City from February to April, 1989–2012. Time-series analyses were performed to estimate the clinic-specific relative risk (RR) of clinical pollinosis associated with AD, adjusting for airborne pollen, suspended particulate matter (SPM), meteorological and temporal factors. Delayed effects were considered. The association with SPM was also examined given its relationship with AD. The clinic-specific RRs were combined using meta-analytic technique.

**Results:**

AD on the same day (lag 0) and the previous 3 to 5 days (lags 3, 4, and 5) was positively associated with the risk of medical consultations for pollinosis. Clinic visits were 21.5% (95% confidence interval 3.1% – 43.1%) higher when there was an AD event (across lags 0–5). The association with SPM showed comparable lag structure, but with smaller effect estimates. When stratified by the occurrence of AD, the estimated risk increases associated with SPM did not differ between the AD-affected and AD-free days.

**Conclusion:**

AD is associated with an increased risk of medical consultations for pollinosis in spring. More research is needed to elucidate the roles of air particles with different sizes.

**Electronic supplementary material:**

The online version of this article (doi:10.1186/s12199-017-0623-x) contains supplementary material, which is available to authorized users.

## Background

Asian Dust (AD) is a meteorological event that affects Japan especially in the spring when dusts are transported eastward via airstreams from the deserts of China and Mongolia. A number of studies have suggested that AD can adversely impact human health. Specifically, earlier research has linked AD to an increased risk of all-cause [1–4], cardiovascular [1, 5, 6], cerebrovascular [3] and respiratory mortality [7, 8]. Several studies have also indicated that the risk of hospitalization for respiratory [9, 10], cerebrovascular [11] and cardiovascular diseases [10, 12] may increase on AD days.

There is some evidence that AD may also contribute to the worsening of allergic disease symptoms [13–15], although this association has not been consistently observed in previous studies [16–18]. In Japan, severe AD events have been found to be associated with an increased risk of hospitalization for asthma [13] and aggravated lower respiratory symptoms [14]. On the other hand, a study using data from Fukuoka in Kyushu Japan showed that the odds of asthma hospitalization among children for a 10 μg/m^3^ increase in SPM did not differ between the AD and non-AD days, suggesting a lack of association between children asthma hospitalization and AD events [16]. Non-significant associations have also been reported in a study of daily asthmatic admissions in Taipei [17] and daily clinical consultations for conjunctivitis in the same location [18].

To our best knowledge, no previous study has examined if AD might potentially contribute to an increase in the number of medical consultations for pollinosis. This is an important research gap, especially since there have been a few small-size studies (*n* = 23 to 54) which indicated that allergic symptoms such as allergic rhinitis – a main symptom of pollinosis – might be aggravated by AD [19–21]. For example, a cross-sectional study has suggested that AD might act as an adjuvant in promoting allergic rhinoconjunctivitis induced by the inhalation of allergens such as pollens and fungi [19]. Another study has reported that nasal and ocular allergic symptoms are associated with AD before and after the Japanese cedar pollen season, but not during [20]. The worsening of nasopharyngeal, ocular, respiratory, and skin allergic symptoms have also been observed on AD event days in a study in Yonago, a city facing the Sea of Japan [21].

**Fig. 1:**
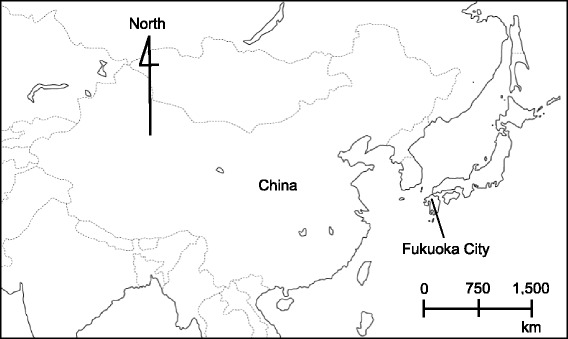
Location of Fukuoka City in East Asia

The objective of the current study is to examine the association between airborne AD and the daily number of medical consultations for pollinosis. We used data from Fukuoka City, which is located in the Kyushu region of Japan close to the Eurasian continent with frequent experiences of AD events in the spring (Fig. 1). The associations of health problems, such as asthmatic symptoms and ischemic stroke with AD have been previously investigated in this location [16, 22]. In the past, some studies have relied on PM_10_/PM_2.5_ to ascertain the occurrence of AD [1, 17, 18, 23]. However, recently it has been proposed that AD may serve as an aid to enhance allergic reactions independent of particulate matter [13–15, 19–21]. Hence, in the present study, an AD event was defined based on the criteria set by the Japan Meteorological Agency, i.e., when any of the air monitoring stations located in Fukuoka City reported a turbid atmosphere, i.e., visibility < 10 km, or when AD particles are visible via remote sensing or sun photometer. We also included the suspended particulate matter (SPM) in the current study in view of its relationship with AD (i.e. higher SPM level on AD days) [16], and controlled for airborne pollen level as the main trigger of pollinosis in all analyses. Assuming that the exacerbation of pollinosis symptoms by atmospheric environment is reflected in the daily number of medical consultation for pollinosis [24], we analyzed data comprising 24 years of daily consultations for pollinosis and the environmental factors (i.e. AD and SPM) in Fukuoka City using time series regression.

## Methods

### Clinic visit data

**Fig. 2:**
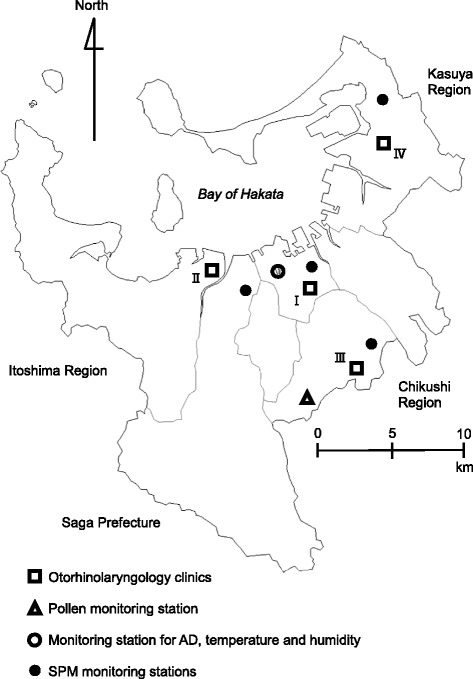
Fukuoka City and the location of the otorhinolaryngology clinics and monitoring stations for air pollen, AD, SPM and weather variables

Data on the daily number of consultations for pollinosis at 4 otorhinolaryngology clinics (referred to as Clinic I, II, III, and IV) in Fukuoka City, Japan were obtained from the database organized by the Fukuoka Prefecture Medical Association and the Fukuoka Prefecture Medical Center [25] (Fig. 2). From 13 clinics listed in the database these 4 clinics were selected based on their availability of the daily consultation data and their proximity to where the airborne pollen and air pollutants were measured. The database contains the daily number of medical consultations for pollinosis at each clinic and contains no personal identifiable information of patients. Pollinosis cases were diagnosed according to the clinical practice guidelines for allergic rhinitis at each clinic [26]. The period of data varied by clinic: 1989 to 2012 for Clinic I and III, 1994 to 2012 for Clinic II, and 1989 to 2001 for Clinic IV.

### Pollen data

Daily measurements of airborne pollen were obtained from the National Hospital Organization Fukuoka Hospital in Fukuoka City (Fig. 2). The ambient concentrations of pollen from Cryptomeria japonica (Japanese cedar or *sugi* in Japanese) and Chamaecyparis obtusa (Japanese cypress or *hinoki* in Japanese) were determined using the Durham technique [27], whereby the airborne pollen grains were captured on a vaseline coated glass slide fixed inside a Durham sampler. Pollen grains on the glass slide were then stained with fuchsin and counted. The slides were changed every day except on weekends where they were left for 2 to 3 days before retrieval. For the analysis, the 2- or 3-day averages were computed for pollen counts on weekends (e.g., the same values for Friday, Saturday, and Sunday if only a single pollen count was provided for a particular weekend). We combined *sugi* and *hinoki* pollen counts in the analysis as both are known to induce pollinosis from February to April [26]. The pollen data used in the analysis were cubic root transformed in order to improve the normality of its distribution.

### Environmental data

We obtained the AD data from the Japan Meteorological Agency (JMA). AD was monitored daily at the Fukuoka District Meteorological Observatory in Fukuoka City and recorded as either present or absent based on visual observation that took place at the observatory (Fig. 2) at random time every day. The JMA defines AD day based on a visibility criterion of <10 km as well as the information from sun photometer or satellites [28].

For air pollution data, continuous air quality monitoring stations were identified based on their proximity to the clinics (Fig. 2). We extracted the hourly concentrations of SPM from a database provided by the National Institute for Environmental Studies, Japan. SPM is defined as particle with aerodynamic diameter of less than 10 μm at 100% efficiency cut-off [29]. The theoretical 50% cut-off diameter for SPM is assumed to be approximately 7 μm [29]. We computed the daily mean concentration of SPM from the hourly data provided when there were no more than 5 missing hourly values per day. The daily SPM levels were highly correlated among the stations, with correlation coefficients ranging between 0.80 and 0.89. The daily values from the 4 stations were therefore averaged for analysis.

Data on daily mean temperature (°C) and relative humidity (%) measured continuously at Chuo-ku, Fukuoka City was also obtained from the JMA for confounding adjustment.

### Statistical analysis

We analyzed data from February to April using a generalized linear model with quasi-Poisson link to account for overdispersion in the outcome – the daily number of clinic visits for pollinosis. The exposure variables were the presence/absence of AD and the daily mean concentrations of SPM. Other variables included in the model were the day of the week (indicator denoting Monday to Sunday), public holidays (yes/no), month (indicator for January to December), year indicator, pollen (average of lags 0–5, i.e., mean concentration on the current and 5 previous days) and the natural cubic splines of daily mean temperature and relative humidity with 3° of freedom. The choice of the 5-days delayed effects of pollen was based on findings from a previous study in Tokyo [24].

We computed the relative risk (RR) and the corresponding 95% confidence intervals (CI) for the association between the daily consultations for pollinosis and the occurrence of AD for each clinic using single- and bi-pollutant models with the addition of SPM. Both models included a term for the interaction between AD and pollen. The bi-pollutant models additionally accounted for the interaction between AD and SPM, and they provide SPM-adjusted effect estimates of AD, which allow further examination of the AD-pollinosis association excluding the portion attributable to SPM. We examined the single-day delayed effects of AD from lag 0 (current day) to lag 6 one at a time (because of correlated lag terms). We then computed the cumulative effects using exposure information up to the previous 5 days (i.e. combining the indicators across lags 0–5 and excluding lag 6 where the association began to attenuate). To examine the association between SPM and AD days, the effect of SPM was estimated in the same manner stratified by AD-affected or AD-free days, with consideration for the interaction between SPM and pollen. The statistical difference of the estimates for the effects of SPM on AD-affected days and AD-free days was tested using the confidence interval method [30].

To obtain an average effect of AD or SPM for the study location, we pooled the clinic-specific estimates using a random effects model. The RR estimates for the effects of SPM correspond to a 10 μg/m^3^ increase in the mean concentration of the pollutant. Confidence intervals that exclude 1 indicate statistically significant effect estimates. We performed all analyses in R (version 2.15.3; R Project for Statistical Computing, Vienna, Austria). The level of statistical significance was set at *p* < 0.05.

## Results

AD was observed on 238 (6.6%) of the 3630 days included in the study. AD occurred with a median value of 7 days per year between February and April. A total of 65,488 clinic visits for pollinosis were analyzed. Across the 4 clinics, there were 9.2 visits per day during the 3-months period. The frequency of visits did not differ statistically between AD-affected and AD-free days (Table 1). The level of SPM was significantly higher when AD was present, while the concentration of pollen was only marginally higher. Temperature and relative humidity differed significantly based on the presence of AD.

**Table 1:** Summary statistics for daily clinic consultations, concentrations of SPM, airborne pollen and meteorological variables by the presence of AD

	AD-affected days	AD-free days	*P*-value for difference
Total number of days	238	3392	
Median (range) per year	7 (1, 26)	82 (63, 89)	
Clinic consultation [mean (SD)]			
Clinic I	8.4 (9.9)	8.7 (10.4)	0.65
Clinic II	7.7 (8.7)	8.8 (9.0)	0.14
Clinic III	11.9 (12.4)	12.3 (13.3)	0.73
Clinic IV	4.6 (7.0)	5.2 (6.8)	0.39
SPM (μg/m^3^)			
Mean (SD)	58.9 (32.2)	29.4 (13.4)	<0.01
Median (IQR)	52.7 (40.2, 67.6)	26.8 (19.8, 36.0)	
10th, 90th centile	28.6, 88.3	15.3, 47.1	
Days missing (%)^a^	10 (1.5)	174 (2.7)	
Pollen (grain/cm^2^)			
Mean (SD)	45.5 (110.4)	30.1 (77.2)	0.06
Median (IQR)	3.5 (0.8, 23.1)	3.0 (0.5, 18.5)	
10th, 90th centile	0, 153	0, 90	
Days missing (%)^a^	0 (0)	53 (0.8)	
Temperature (°C)			
Mean (SD)	13.9 (3.2)	11.0 (4.2)	<0.01
Relative humidity (%)			
Mean (SD)	58.3 (10.7)	63.5 (13.1)	<0.01

*AD* Asian dust, *SPM* suspended particulate matter, *SD* standard deviation, *IQR* interquartile range

^a^Total for four clinics

AD was associated with increased risk of clinic visits for pollinosis at lag 0 (pooled RR = 1.175, 95% CI 1.061–1.301, Table 2). The estimated risk increased gradually until lag 4, with lags 3 to 5 being statistically significant before attenuation at lag 6. The 6-days cumulative RR (lags 0–5) was 1.215 (95% CI 1.031–1.431). In the bi-pollutant model, the estimated RR for the effect of AD increased slightly when SPM was added to the model. The estimated effects of SPM and its interaction with AD were small and generally insignificant (Table 2 and Additional file 1: Table S1).

**Table 2:** Relative risks ^a^ and 95% confidence intervals for daily medical consultations for pollinosis in association with exposure to AD

Lag period for AD	AD only (Single-pollutant model)			AD with SPM ^b^ (Bi-pollutant model)			
	RR ^c^	95% CI		RR ^c^	95% CI		*P*-value for AD × SPM
Clinic I (*n* = 18571)							
Lag 0	1.215	1.003	1.472	1.383	1.046	1.828	0.43
Lag 1	1.353	1.116	1.640	1.814	1.348	2.442	0.05
Lag 2	1.284	1.053	1.566	1.850	1.361	2.516	0.01
Lag 3	1.362	1.130	1.642	1.628	1.201	2.206	0.31
Lag 4	1.404	1.160	1.700	1.897	1.406	2.559	0.05
Lag 5	1.211	0.991	1.479	1.707	1.250	2.331	0.01
Lag 6	1.315	1.078	1.605	1.814	1.353	2.433	>0.01
Lags 0–5	1.241	1.095	1.407	1.440	1.175	1.766	0.31
Clinic II (*n* =14741)							
Lag 0	1.344	1.316	1.373	1.107	0.863	1.421	0.70
Lag 1	0.948	0.779	1.155	1.022	0.762	1.370	0.93
Lag 2	0.925	0.765	1.118	1.011	0.759	1.346	0.67
Lag 3	1.080	0.905	1.288	1.222	0.917	1.629	0.57
Lag 4	1.082	0.905	1.293	1.207	0.908	1.605	0.57
Lag 5	1.067	0.889	1.280	1.122	0.851	1.479	0.77
Lag 6	0.890	0.739	1.072	1.001	0.770	1.301	0.21
Lags 0–5	1.029	0.919	1.152	1.120	0.933	1.345	0.62
Clinic III (*n* = 26186)							
Lag 0	1.177	0.993	1.394	1.119	0.879	1.426	0.37
Lag 1	1.126	0.944	1.344	1.049	0.804	1.370	0.37
Lag 2	1.214	1.019	1.445	1.196	0.916	1.560	0.82
Lag 3	1.276	1.079	1.509	1.273	0.971	1.669	0.75
Lag 4	1.241	1.047	1.470	1.200	0.923	1.559	0.47
Lag 5	1.192	1.005	1.415	1.151	0.891	1.488	0.53
Lag 6	1.142	0.961	1.358	1.179	0.922	1.509	0.78
Lags 0–5	1.147	1.025	1.283	1.167	0.975	1.397	0.90
Clinic IV (*n* = 5990)							
Lag 0	1.506	1.101	2.060	1.442	0.891	2.332	0.83
Lag 1	1.734	1.288	2.334	2.177	1.342	3.533	0.25
Lag 2	1.769	1.286	2.434	2.299	1.384	3.820	0.16
Lag 3	1.668	1.216	2.288	1.820	1.090	3.038	0.58
Lag 4	1.761	1.275	2.430	1.646	1.002	2.706	0.80
Lag 5	1.181	0.851	1.639	1.244	0.748	2.069	0.76
Lag 6	1.364	0.980	1.899	1.655	0.999	2.741	0.27
Lags 0–5	1.594	1.283	1.980	2.024	1.408	2.910	0.07
Pooled estimates							
Lag 0	1.175	1.061	1.301	1.203	1.045	1.385	-
Lag 1	1.238	0.976	1.570	1.395	0.970	2.006	-
Lag 2	1.238	0.972	1.576	1.456	1.021	2.075	-
Lag 3	1.290	1.111	1.497	1.391	1.184	1.634	-
Lag 4	1.309	1.099	1.559	1.427	1.122	1.816	-
Lag 5	1.156	1.045	1.279	1.278	1.039	1.572	-
Lag 6	1.142	0.943	1.383	1.337	1.006	1.777	-
Lags 0–5	1.215	1.031	1.431	1.348	1.075	1.690	-

*AD* Asian dust, *SPM* suspended particulate matter, *RR* relative risk, *CI* confidence interval

^a^Using single-lag model with adjustment for the day of the week, public holidays, month, year, pollen (average lag 0–5), interaction between AD-pollen and the natural cubic splines of daily mean temperature and relative humidity with 3° of freedom

^b^Additional adjustment for SPM (average lags 0–5) and the corresponding interaction between AD-SPM; refer to Additional file 1: Table S1 for further information

^c^Reference: AD-free days

To further examine the effect of SPM given the higher concentration during the AD events, we stratified the effect of SPM into AD-affected and AD-free days (Table 3). The estimated 6-days cumulative effect of SPM (lag 0–5) was slightly larger on AD-affected days than on AD-free days (RR = 1.066, 95% CI 1.007–1.127 vs. RR = 1.062, 95% CI 1.022–1.104), although the difference was not statistically significant. When Clinic IV was excluded because of the shorter study period, the difference remained statistically insignificant. The estimated effects of SPM were delayed for approximately 5 days and attenuated at lag 6 on both the AD-affected and AD-free days (Table 3). The breakdown by clinic is presented in Additional file 2: Table S2.

**Table 3:** Pooled estimates for the relative risk^a^ with 95% confidence intervals of daily medical consultations for pollinosis in association with a 10-μg/m^3^ increase in mean suspended particulate matter, by the presence of AD

Lag period for SPM^b^	(A) AD-affected days			(B) AD-free days			*P*-value for (A) – (B)
	RR	95% CI		RR	95% CI		
Lag 0	0.972	0.931	1.015	1.004	0.983	1.025	0.19
Lag 1	1.000	0.963	1.039	1.005	0.986	1.025	0.84
Lag 2	1.032	0.996	1.070	1.017	0.998	1.037	0.49
Lag 3	1.028	0.988	1.071	1.038	1.018	1.059	0.69
Lag 4	1.038	1.001	1.076	1.026	1.010	1.042	0.58
Lag 5	1.060	1.011	1.111	1.023	1.007	1.038	0.15
Lag 6	0.999	0.942	1.060	1.014	0.993	1.035	0.66
Lags 0–5							
All clinics	1.066	1.007	1.127	1.062	1.022	1.104	0.94
Except Clinic IV	1.071	1.011	1.135	1.055	1.014	1.097	0.68

*AD* Asian Dust, *SPM* suspended particulate matter, *RR* relative risk, *CI* confidence interval

^a^Using single-lag model with adjustment for the day of the week, public holidays, month, year, the natural cubic splines of daily mean temperature, relative humidity and pollen (lag 0–5) with 3° of freedom. An interaction term between SPM and pollen concentration was included. Refer to Additional file 2: Table S2 for further information

^b^Delayed effects longer than 1 day were constrained as average

## Discussion

Findings of the current study suggest for the first time that AD is associated with an increased number of daily medical consultations for pollinosis in Japan. The number of consultations increased by approximately 21.5% (95%CI 3.1% – 43.1%) on average across the four clinics when there was an AD event (Table 2). The association was short-term beginning immediately at lag 0 (i.e. on the same day) and observed up to the previous 5 days. When SPM was included as adjustment, the estimates of AD effect remained significant (but they increased slightly likely because of collinearity). We did not find evidence of interaction between AD and SPM (Additional file 1: Table S1), despite the higher SPM level on AD days. The results of further analysis showed that SPM was associated with the daily consultations for pollinosis, with a lag structure that was fairly similar to that of AD (Table 3). The estimated effects of SPM were smaller than those for AD, and they did not differ between the AD and non-AD days, which has also been reported in a study of asthma admission among children and AD in the same location [16].

There are two possible ways AD might be linked to the exacerbation of pollinosis symptoms, which might in turn leads to an increase in the number of consultations. First, AD may act as an adjuvant for allergens [31]. AD particles contain soil-derived metals, anthropogenic metals, and other forms of atmospheric pollutants by human activity, and then act to promote nasal and ocular symptoms [31]. Second, AD components may contain bacteria, fungi and viruses, all of which can have a direct effect as allergens [3, 19, 32]. For example, earlier research has suggested that desert dust may contain fungi which are allergenic, [33], and that airborne bacteria may increase by approximately 4 times on AD days compared with non-AD days [32]. It has been suggested that these microorganisms may negatively affect the human immune system and exacerbate pollinosis symptoms [33, 34].

We observed a delay of less than a week (i.e. until lag 5) in the association between AD and the daily consultations for pollinosis in this study. This delayed effect was noted in all the clinics except Clinic II (Table 2). A possible explanation is that some patients were likely not able to visit the clinics on the same day when their symptoms became worse, and their visits later were registered as a lag effect [16]. It is also possible that the transported dust remains in the air for several days after an AD event even if it is not observed visually, although further investigation is required to understand this phenomenon, particularly the role of long-range fine/ultrafine particles that can remain suspended in the air for a longer period.

In this study, we examined the effect of AD defined based on meteorological conditions instead of the level of SPM. This allows us to examine the total effect of AD, which may encompass contribution by factors unrelated to SPM (e.g., microorganism-related AD factor described above). Our results showed that the effect estimates for AD remained significant after adjusting for SPM, implying there might be other air pollutants or unmeasured exposures that might have triggered or worsened the pollinosis symptoms on AD days. Besides the microscopic organisms, a likely explanation would be that AD might also represent smaller inhalable particles such as fine particulate matter (PM_2.5_). It has been previously suggested that the aeroallergens from pollens might be able to bind to small air particles [35], and in Japan, a study has indicated that particles as fine as 0.3 μm might contain allergens such as Cry j 1 and Cry j 2 from the Japanese cedar pollens [36]. In addition, a recent study has indicated that the synergetic effect between airborne pollen and PM_2.5_ was stronger and more robust than the interaction between pollen and SPM [24]. Nevertheless, because the measurements of PM_2.5_ were not available for most of the study period (the data was only available since 2010), we were unable to examine this in the context of AD and pollinosis consultations. Future studies are therefore needed to explore the roles of air particles of different size, including their origin (e.g. transported or local), chemical constituents, and potential interactions with airborne pollen concentrations during the spring season.

This study has several limitations. First, we had no information on age, sex and individual exposure or about the participants’ pollinosis history or history of other allergic diseases. The association between the daily number of medical consultations and AD might differ across such individual characteristics. Second, it was not possible to separate the first clinic visits from re-visits in the same year. First visit refers to the first examination that occurs when a patient start to experience pollinosis symptoms in each pollen season, while re-visits are usually re-examinations for the purpose of medicine refill. In the present study, as data with separate first and re-visits were not available, it is possible the association between air pollen and clinic visits for pollinosis might have been underestimated, leading to incorrect estimates for AD and SPM. For example, two previous studies that used data from a clinic in Tokyo reported that the positive correlation between the pollen count and the number of first examinations for pollinosis was stronger than that for re-examinations [34, 37], suggesting that the first visits might better reflect the severity of pollinosis symptoms. Third, this study did not consider other criteria air pollutants such as sulfur dioxide, nitrogen dioxide and ozone, which require further investigations as there have been evidence linking air pollution and the prevalence of allergic rhinitis [38, 39].

## Conclusions

AD is positively associated with the daily number of medical consultations for pollinosis in the spring season. The increase in risk began on the day of AD and was attenuated within a week. The underlying mechanisms through which AD exacerbates the symptoms of pollinosis should be examined in future research.

## Additional file

Additional file 1: Table S1. Regression^a^ coefficients (beta), standard errors (SE) and *P* - values for the Asian dust (AD) indicator variable, pollen concentration, interaction between AD and pollen, suspended paticulate matter (SPM) and interaction between AD and SPM, by clinic in Fukuoka City, Japan. (PDF 233 kb)
Additional file 2: Table S2. Regression^a^ coefficients (beta), standard errors (SE) and *P* - values for suspended paticulate matter (SPM), pollen concentration, and their interaction for four clinics in Fukuoka City, Japan. (PDF 179 kb)

## Abbreviations

AD: Asian Dust
CI: Confidence intervals
RR: Relative risk
SPM: Suspended particulate matter
