# Supplementary material for: Association of Asian Dust with daily medical consultations for pollinosis in Fukuoka City, Japan
Source: Environ Health Prev Med. 2017 Apr 4;22:25. doi: 10.1186/s12199-017-0623-x (PMC5664566; doi:10.1186/s12199-017-0623-x)
Supplement: Additional file 1: Table S1. — Regressiona coefficients (beta), standard errors (SE) and P - values for the Asian dust (AD) indicator variable, pollen concentration, interaction between AD and pollen, suspended paticulate matter (SPM) and interaction between AD and SPM, by clinic in Fukuoka City, Japan. (PDF 233 kb) [file 12199_2017_623_MOESM1_ESM.pdf]

Supplementary Data

Table S1

Regression[a] coefficients (beta), standard errors (SE) and *P*-values for the Asian dust (AD) indicator variable, pollen concentration, interaction between AD and pollen, suspended particulate matter (SPM) and interaction between AD and SPM, by each clinic in Fukuoka City, Japan.

Clinic and lag	AD			Pollen[c]			AD × Pollen interaction		
period for AD[b]	beta	SE	*P*-value	beta	SE	*P*-value	beta	SE	*P*-value
Clinic I									
Lag 0	0.324	0.143	0.02	0.302	0.011	< 0.01	-0.063	0.027	0.02
Lag 1	0.596	0.152	< 0.01	0.308	0.011	< 0.01	-0.116	0.026	< 0.01
Lag 2	0.615	0.157	< 0.01	0.302	0.011	< 0.01	-0.080	0.028	< 0.01
Lag 3	0.487	0.155	< 0.01	0.312	0.011	< 0.01	-0.124	0.026	< 0.01
Lag 4	0.640	0.153	< 0.01	0.314	0.011	< 0.01	-0.137	0.025	< 0.01
Lag 5	0.535	0.159	< 0.01	0.309	0.011	< 0.01	-0.100	0.027	< 0.01
Lag 6	0.596	0.150	< 0.01	0.308	0.011	< 0.01	-0.115	0.029	< 0.01
Lag 0-5	0.365	0.104	< 0.01	0.324	0.012	< 0.01	-0.086	0.018	< 0.01
Clinic II									
Lag 0	0.102	0.127	0.42	0.179	0.010	< 0.01	-0.029	0.025	0.24
Lag 1	0.022	0.150	0.89	0.180	0.010	< 0.01	-0.026	0.025	0.30
Lag 2	0.011	0.146	0.94	0.177	0.010	< 0.01	-0.009	0.026	0.74
Lag 3	0.201	0.146	0.17	0.185	0.010	< 0.01	-0.069	0.024	< 0.01
Lag 4	0.189	0.145	0.19	0.186	0.010	< 0.01	-0.070	0.023	< 0.01
Lag 5	0.115	0.141	0.41	0.183	0.010	< 0.01	-0.056	0.025	0.02
Lag 6	0.001	0.134	0.99	0.178	0.010	< 0.01	-0.003	0.025	0.90
Lag 0-5	0.114	0.093	0.22	0.189	0.011	< 0.01	-0.039	0.017	0.02
Clinic III									
Lag 0	0.113	0.124	0.36	0.289	0.010	< 0.01	-0.062	0.024	< 0.01
Lag 1	0.048	0.136	0.72	0.291	0.010	< 0.01	-0.080	0.024	< 0.01
Lag 2	0.179	0.136	0.19	0.291	0.010	< 0.01	-0.090	0.025	< 0.01
Lag 3	0.241	0.138	0.08	0.296	0.010	< 0.01	-0.103	0.023	< 0.01
Lag 4	0.182	0.134	0.17	0.297	0.010	< 0.01	-0.096	0.022	< 0.01
Lag 5	0.141	0.131	0.28	0.293	0.010	< 0.01	-0.077	0.023	< 0.01
Lag 6	0.165	0.126	0.19	0.290	0.010	< 0.01	-0.063	0.023	< 0.01
Lag 0-5	0.155	0.092	0.09	0.306	0.011	< 0.01	-0.071	0.016	< 0.01
Clinic IV									
Lag 0	0.366	0.245	0.14	0.381	0.021	< 0.01	-0.135	0.047	< 0.01
Lag 1	0.778	0.247	< 0.01	0.391	0.021	< 0.01	-0.153	0.044	< 0.01
Lag 2	0.833	0.259	< 0.01	0.390	0.021	< 0.01	-0.152	0.047	< 0.01
Lag 3	0.599	0.261	0.02	0.398	0.021	< 0.01	-0.178	0.043	< 0.01
Lag 4	0.498	0.254	0.05	0.408	0.021	< 0.01	-0.227	0.044	< 0.01
Lag 5	0.218	0.260	0.40	0.399	0.021	< 0.01	-0.153	0.045	< 0.01
Lag 6	0.504	0.258	0.05	0.399	0.021	< 0.01	-0.164	0.048	< 0.01
Lag 0-5	0.705	0.185	< 0.01	0.429	0.023	< 0.01	-0.157	0.032	< 0.01

[a] Adjusted for the day of the week, public holidays, month, year, and the natural cubic splines of daily mean temperature and relative humidity with 3 degrees of freedom.

[b] Delayed effects longer than a day were constrained as mean.

[c] Cubic-root transformed.

Table S1 cont.

Clinic and lag period for AD[b]	SPM			AD × SPM interaction		
	beta	SE	*P*-value	beta	SE	*P*-value
Clinic I						
Lag 0	-0.003	0.002	0.04	-0.002	0.003	0.43
Lag 1	-0.002	0.002	0.33	-0.006	0.003	0.05
Lag 2	-0.002	0.002	0.21	-0.008	0.003	0.01
Lag 3	-0.003	0.002	0.09	-0.003	0.003	0.31
Lag 4	-0.002	0.002	0.25	-0.006	0.003	0.05
Lag 5	-0.001	0.002	0.47	-0.009	0.003	0.01
Lag 6	-0.002	0.002	0.24	-0.010	0.004	< 0.01
Lags 0-5	-0.002	0.002	0.25	-0.003	0.003	0.31
Clinic II						
Lag 0	-0.003	0.001	0.05	-0.001	0.003	0.70
Lag 1	-0.003	0.002	0.08	-0.000	0.003	0.93
Lag 2	-0.003	0.001	0.09	-0.001	0.003	0.67
Lag 3	-0.002	0.001	0.10	-0.002	0.003	0.57
Lag 4	-0.002	0.001	0.12	-0.002	0.003	0.57
Lag 5	-0.003	0.001	0.08	-0.001	0.003	0.77
Lag 6	-0.002	0.001	0.08	-0.004	0.003	0.21
Lags 0-5	-0.002	0.002	0.24	-0.001	0.002	0.62
Clinic III						
Lag 0	-0.002	0.001	0.09	0.002	0.003	0.37
Lag 1	-0.001	0.002	0.34	0.002	0.003	0.37
Lag 2	-0.001	0.001	0.48	0.001	0.003	0.82
Lag 3	-0.002	0.001	0.26	0.001	0.003	0.75
Lag 4	-0.002	0.001	0.17	0.002	0.003	0.47
Lag 5	-0.002	0.001	0.15	0.002	0.003	0.53
Lag 6	-0.002	0.001	0.21	-0.001	0.003	0.78
Lags 0-5	-0.001	0.002	0.52	0.000	0.002	0.90
Clinic IV						
Lag 0	-0.004	0.003	0.21	0.001	0.006	0.83
Lag 1	-0.001	0.003	0.72	-0.007	0.006	0.25
Lag 2	0.000	0.003	0.99	-0.009	0.006	0.16
Lag 3	-0.000	0.003	0.95	-0.003	0.006	0.58
Lag 4	-0.001	0.003	0.73	0.001	0.006	0.80
Lag 5	-0.001	0.003	0.73	-0.002	0.007	0.76
Lag 6	-0.003	0.003	0.25	-0.008	0.007	0.27
Lags 0-5	0.003	0.004	0.39	-0.009	0.005	0.07
