# Supplementary material for: Association of Asian Dust with daily medical consultations for pollinosis in Fukuoka City, Japan
Source: Environ Health Prev Med. 2017 Apr 4;22:25. doi: 10.1186/s12199-017-0623-x (PMC5664566; doi:10.1186/s12199-017-0623-x)
Supplement: Additional file 2: Table S2. — Regressiona coefficients (beta), standard errors (SE) and P - values for suspended paticulate matter (SPM), pollen concentration, and their interaction for four clinics in Fukuoka City, Japan. (PDF 179 kb) [file 12199_2017_623_MOESM2_ESM.pdf]

Table S2

Regression[a] coefficients (beta), standard errors (SE) and *P*-values for suspended particulate matter (SPM), pollen concentration, and their interaction for four clinics in Fukuoka City, Japan.

	SPM			Pollen[c]			SPM × Pollen interaction		
SPM (Lags 0 – 5)[b]	beta	SE	*P*-value	beta	SE	*P*-value	beta	SE	*P*-value
Asian-dust affected days									
Clinic I	0.0096	0.0053	0.07	0.4021	0.0811	< 0.01	-0.0042	0.0014	< 0.01
Clinic II	0.0067	0.0056	0.23	0.2242	0.0905	0.01	-0.0020	0.0014	0.17
Clinic III	0.0049	0.0046	0.29	0.2435	0.0704	< 0.01	-0.0002	0.0012	0.85
Clinic IV	-0.0045	0.0136	0.74	0.0444	0.1726	0.80	0.0014	0.0030	0.63
Pooled	0.0064	0.0029	0.03	0.2672	0.0559	< 0.01	-	-	-
Asian-dust-free days									
Clinic I	0.0076	0.0028	< 0.01	0.4479	0.0317	< 0.01	-0.0042	0.0009	< 0.01
Clinic II	0.0017	0.0024	0.48	0.2457	0.0273	< 0.01	-0.0021	0.0008	< 0.01
Clinic III	0.0072	0.0026	< 0.01	0.4178	0.0283	< 0.01	-0.0038	0.0008	< 0.01
Clinic IV	0.0126	0.0057	0.03	0.5782	0.0573	< 0.01	-0.0057	0.0016	< 0.01
Pooled	0.0061	0.0020	< 0.01	0.4172	0.0671	< 0.01	-	-	-

[a] Adjusted for the day of the week, public holidays, month, year, and the natural cubic splines of daily mean temperature and relative humidity with 3 degrees of freedom.

[b] Constrained as mean.

[c] Cubic-root transformed.
